# Antidepressant-Like and Antioxidant Effects of *Plinia trunciflora* in Mice

**DOI:** 10.1155/2015/601503

**Published:** 2015-07-02

**Authors:** Cassia Sacchet, Ricieri Mocelin, Adrieli Sachett, Fernanda Bevilaqua, Rafael Chitolina, Fernanda Kuhn, Aline Augusti Boligon, Margareth Linde Athayde, Walter Antonio Roman Junior, Denis Broock Rosemberg, Jacir Dal Magro, Greicy Michelle Marafiga Conterato, Angelo L. Piato

**Affiliations:** ^1^Programa de Pós-Graduação em Ciências Ambientais, Unochapecó, Avenida Senador Attílio Fontana 591E, 89809-000 Chapecó, SC, Brazil; ^2^Núcleo de Fitoterápicos, Programa de Pós-Graduação em Ciências da Saúde, Unochapecó, Avenida Senador Attílio Fontana 591E, 89809-000 Chapecó, SC, Brazil; ^3^Laboratório de Fitoquímica, Universidade Federal de Santa Maria, Avenida Roraima 1000, 97105-900 Santa Maria, RS, Brazil; ^4^Programa de Pós-Graduação em Bioquímica Toxicológica, Universidade Federal de Santa Maria, Avenida Roraima 1000, 97105-900 Santa Maria, RS, Brazil; ^5^Laboratório de Fisiologia da Reprodução Animal, Universidade Federal de Santa Catarina, Rodovia Ulisses Gaboardi, Km 3, Campus Curitibanos, 89520-000 Curitibanos, SC, Brazil; ^6^Programa de Pós-Graduação em Farmacologia e Terapêutica, Universidade Federal do Rio Grande do Sul, Avenida Sarmento Leite 500/305, 90050-170 Porto Alegre, RS, Brazil

## Abstract

The jaboticaba tree, *Plinia trunciflora* (O. Berg) Kausel, is popularly named “jabuticabeira” in Brazil and is used in folk medicine to treat diabetes and chronic inflammation of the tonsils, but studies evaluating the central effects of this species are limited. This study evaluated the antidepressant-like and antioxidant effects of *P. trunciflora* (PT) aqueous extract, in which five different anthocyanins were identified. PT showed significant ferric-reduction power and DPPH radical scavenging activity *in vitro* and reduced lipid peroxidation both *in vitro* and *ex vivo*. At the behavioural level, PT (400 and 800 mg/kg, i.p.) dose-dependently reduced immobility time in the tail suspension test in Swiss male mice. The identification of bioactive compounds accompanied by the *in vitro* and *ex vivo* antioxidant activity of PT suggests that these activities might be related to the antidepressant-like activity of *P. trunciflora*.

## 1. Introduction

Depression is a common, serious, and recurrent chronic affective disorder characterized by anhedonia, headache, sleep disturbances, changes in sexual desire, and a loss of energy [1]. This disease is among the five most prevalent in the world and is expected to be the second leading cause of disability in 2020 [2]. The monoaminergic hypothesis of depression [3] does not provide a full understanding of the progression, causes, and pharmacotherapy of depression. New hypotheses have been postulated, and oxidative stress has been suggested to be involved in the pathophysiology of depression [4].

Oxidative stress is a condition in which an imbalance between the production of free radicals and endogenous antioxidant defenses occurs [5], culminating in decreased cell antioxidant capacity. The superoxide anion (O_2_^•−^) and hydrogen peroxide (H_2_O_2_) produced during respiratory chain may generate the highly deleterious hydroxyl radical (^•^OH) via the Fenton reaction [6]. The overproduction of these species is related to protein, DNA, and lipid oxidation [7] as well as the inactivation of important antioxidant enzymes, such as catalase (CAT), superoxide dismutase (SOD), glutathione peroxidase (GPx), and thioredoxin reductase (TrxR) [5]. The overproduction of ROS and oxidative stress have been implicated in the pathophysiological processes related to various diseases, including Alzheimer's, Parkinson's, anxiety, and depression [8–10]. In this sense, plants emerge as potential alternatives for the treatment of oxidative stress-related diseases, considering that they are important sources of carotenoids, flavonoids, vitamins, and polyphenols.

The Myrtaceae family consists of 4,620 species distributed in 140 genera whose occurrence has been described in subtropical and tropical regions of the world, mainly Central and South America and Australia [11]. The jaboticaba tree,* Plinia trunciflora* (O. Berg) Kausel, a synonym of* Myrciaria trunciflora* O. Berg,* Eugenia cauliflora* O. Berg, and* Myrciaria peruviana* (Poir.) Mattos is popularly named “jabuticabeira” in Brazil (source: http://www.tropicos.org/). In folk medicine, species of* Plinia* have been used to treat various diseases, such as diabetes and chronic inflammation of the tonsils [12]. However, studies that evaluate the effects of this species on the central nervous system (CNS) are scarce in the literature. Therefore, the aim of this study was to evaluate the antidepressant-like effect of* P. trunciflora* aqueous extract in the tail suspension test. The identification of bioactive compounds and the* in vitro* and* ex vivo* antioxidant effects of* P. trunciflora* were investigated in order to establish if the antidepressant-like effect of this plant could be related to these properties.

## 2. Materials and Methods

### 2.1. Plant Material

The whole fruits of* Plinia trunciflora* were collected in Alpestre (RS, Brazil) (27°10′56.82′′S and 53°7′19.55′′O), in September, and taxonomically identified by Marcos Eduardo Guerra Sobral (botanical), where a voucher has been deposited in the university herbarium (number 3302).

### 2.2. Preparation of Aqueous Extract of* P. trunciflora* (PT)

The aqueous extracts of whole fruits were prepared based on the methodology described by Kuskoski et al. [13]. The whole fruit (100 g) of* P. trunciflora* was mixed with 200 mL of distilled water and acidified with concentrated HCl until pH 1.5. After trituration for 1 min, the solution was cooled to 4°C over 12 h to extract the anthocyanins. The solution was then centrifuged, and the supernatant was frozen and further lyophilized. Prior to the* in vitro* and* ex vivo* experiments, the lyophilized material was dissolved in ultrapure water (Milli-Q) at the desired concentrations or doses.

### 2.3. Total Phenolic Compounds (TPC)

The total phenolic compounds (TPC) in the PT were determined according to the method described by Singleton and Rossi [14], which is based on the reduction of the phosphowolframate phosphomolybdate complex by phenolics to a blue product that is measured at 750 nm. The results are expressed as gallic acid equivalents (mg gallic acid equivalents/100 g fresh fruit), and the values are presented as the means of triplicate analysis.

### 2.4. Total Monomeric Anthocyanins (TMA)

The total monomeric anthocyanin (TMA) content was determined using the pH differential method [15]. The anthocyanin content was calculated using the molar absorptivity (*ε*) and molecular weights (MW) of cyanidin 3-*O*-glucoside (*ε* = 26,900 L/mol·cm; MW = 449.2 g/mol). The results are expressed as mg of cyanidin 3-*O*-glucoside equivalents/100 g fresh fruit.

### 2.5. Identification and Quantification of Anthocyanins in PT

#### 2.5.1. Chemical, Apparatus, and General Procedures

All chemicals were of analytical grade. Acetonitrile and formic acid were purchased from Merck (Darmstadt, Germany). Cyanidin chloride, malvidin chloride, cyanidin 3-*O*-glucoside chloride, malvidin 3-*O*-glucoside chloride, and delphinidin 3-*O*-glucoside chloride were acquired from ChromaDex. High performance liquid chromatography (HPLC-DAD) was performed with a Shimadzu Prominence Auto Sampler (SIL-20A) HPLC system (Shimadzu, Kyoto, Japan) equipped with Shimadzu LC-20AT reciprocating pumps connected to a DGU 20A5 degasser with a CBM 20A integrator, SPD-M20A diode array detector, and LC solution 1.22 SP1 software.

#### 2.5.2. Quantification of Compounds by HPLC-DAD

Reverse phase chromatographic analyses were carried out under gradient conditions using a C18 column (4.6 mm × 150 mm) packed with 5 *μ*m diameter particles; the mobile phase was water containing 1% formic acid (A) and acetonitrile (B), and the composition gradient was 13% of B until 10 min and changed to obtain 20%, 30%, 50%, 60%, 70%, 20%, and 10% B at 20, 30, 40, 50, 60, 70, and 80 min, respectively [16]. The* P. trunciflora* aqueous extract and mobile phase were filtered through a 0.45 *μ*m membrane filter (Millipore) and then degassed in an ultrasonic bath prior to use; the aqueous extract was analyzed at a concentration of 20 mg/mL. The flow rate was 0.5 mL/min, the injection volume was 20 *μ*L, and the wavelength was 520 nm. Stock solutions of standards references were prepared in the HPLC mobile phase at a concentration range of 0.030 to 0.250 mg/mL. The chromatography peaks were confirmed by comparing their retention times with those of the reference standards and DAD spectra (300 to 700 nm). All chromatography operations were carried out at ambient temperature and in triplicate [17].

### 2.6. Antioxidant Activity* In Vitro*

#### 2.6.1. Determination of Ferric Reducing Antioxidant Power (FRAP)

The FRAP assay was based on Benzie and Strain [18], by measuring the absorbance of the complex formed between Fe^2+^ and Ferric-2,4,6-tripyridyl-s-triazine (TPTZ) at 593 nm after incubation (37°C/15 min) with PT (10–160 *μ*g/mL). The increase in the absorbance was compared to that induced by ascorbic acid (standard), and the results are expressed as the means of the absorbance of triplicate experiments (*n* = 3).

#### 2.6.2. 1,1-Diphenyl-2-2-picrylhydrazyl Radical Scavenging Assay

The antiradical powers of the different concentrations of PT (10–160 *μ*g/mL) and standard were determined by measuring the decrease in the DPPH absorbance after 24 h in the dark compared to a blank [19]. The same procedure was followed for the ascorbic acid standard. This analysis was carried out in triplicate (*n* = 3), and the results are expressed as the means of % inhibition of the DPPH radical, which was calculated as follows: % inhibition = [(Abs control – Abs sample)/Abs control] × 100. The concentration of PT that could scavenge 50% of the DPPH radical (IC_50_) was calculated via a nonlinear regression analysis using the GraphPad Prism Program version 6.0.

#### 2.6.3. Protection against Lipid Peroxidation

A low-speed supernatant (20 min at 2000 ×g) of brain homogenates (50 mM Tris-HCl, pH 7.5, 1 : 9; w/v) was preincubated at 37°C for 1 h in the presence or absence of 50 *μ*M FeCl_2_, 1 mM H_2_O_2_, PT (10–160 *μ*g/mL), and Tris-HCl 50 mM. Subsequently, the amount of thiobarbituric acid-reactive substances (TBARS) was determined [20]. The inhibitory concentration 50 (IC_50_), which represents the concentration of PT that inhibits 50% of lipid peroxidation, was determined via a nonlinear regression analysis using the GraphPad Prism Program version 6.0.

### 2.7. *In Vivo* Studies

#### 2.7.1. Animals

80 two-month-old male Swiss mice (30–40 g) were obtained from the Bioterism Center of Unochapecó. Seven mice were housed per cage (30 × 19 × 13 cm) and maintained in our own animal facility under controlled environmental conditions (22 ± 1°C, 12 hr light/dark cycle, free access to food (Nuvilab CR1) and water). All procedures were carried out in accordance with institutional policies on the handling of experimental animals (approved by the ethics committee, process 001/2012).

#### 2.7.2. Drugs

Fluoxetine was used as commercial Daforin (Laboratório EMS, SP, Brazil). All drugs were dissolved in saline (NaCl 0.9%). The drugs and saline were administered intraperitoneally (*i.p*.) or orally (*p.o*.) at a constant volume of 0.1 mL/10 g body weight.

#### 2.7.3. Tail Suspension Test (TST)

The TST was used as described by Steru et al. [21]. Mice (*n* = 7–10) were orally treated with vehicle (0.9% saline; w/v) or PT (200, 400 or 800 mg/kg). An additional group was treated with fluoxetine (32 mg/kg,* i.p.*). None of the selected doses modified locomotion in the open field test (data not shown). The mice were submitted to the TST for 30 or 60 min (for* i.p.* and* p.o.* treated groups, resp.) after treatments. After the TST, the animals were euthanized, and their brains were removed immediately in order to assess the oxidative stress parameter* ex vivo*. The* ex vivo* analyses were performed in the PT 800 mg/kg group because the behavioral effects of this dose were comparable to those of fluoxetine.

### 2.8. Antioxidant Activity* Ex Vivo*

#### 2.8.1. Antioxidant Enzymes

The antioxidant activity was assessed* ex vivo* using homogenized mouse brains in 7 volumes of 50 mM Tris buffer (pH 7.4). The homogenate was centrifuged at 3000 ×g and 4°C for 10 min to yield a low-speed supernatant for which all parameters were evaluated. The SOD, CAT, and GPx activity were determined according to Misra and Fridovich [22], Aebi [23], and Paglia and Valentine [24], respectively. The TrxR activity was determined using 5,5′-dithiobis (2-nitrobenzoic acid) (DTNB) and NADPH [25]. Gold (III) chloride trihydrate (500 nM) was used to inhibit the thioredoxin reductase activity [26] and determine the nonthioredoxin reductase DTNB reduction, which was subtracted from the total DTNB reduction in order to obtain the thioredoxin reductase activity. The amount of reduced DTNB was calculated using an absorption coefficient of 13.6 × 103/mol/cm.

#### 2.8.2. Nonprotein Thiol Groups

The low-speed supernatant fraction was mixed with 10% trichloroacetic acid (1 : 1 v/v), followed by the centrifugation and neutralization of the supernatant (to pH 7.5) with 1 M Tris. The nonprotein thiol groups were immediately determined using a standard curve of cysteine [27].

#### 2.8.3. Protein Quantification

The protein content was measured using bovine serum albumin as a standard [28].

#### 2.8.4. Lipid Peroxidation

After the addition of 7.2 mM butylated hydroxytoluene to prevent further oxidation, the supernatant was used to determine the amount of reactive thiobarbituric acid [20]. The samples were extracted with n-butanol, and the reaction product was determined at 535 nm using a standard curve of 1,1,3,3-tetraethoxypropane.

### 2.9. Statistical Analysis

The results (cumulative counts for spontaneous locomotion, time in seconds for immobility, and antioxidant activity* in vitro* and* ex vivo*) are expressed as the mean ± S.E.M. Comparisons between the groups were made by one-way ANOVA followed by Tukey's post hoc test. The differences between the data were analyzed using Student's *t*-test to assess the antioxidant activity* in vitro* (different concentrations of PT × different concentrations of ascorbic acid). Results with *p* < 0.05 were considered significant. The regression analyses were made using Statistica 7.0 software system (Statsoft Inc., 2001).

## 3. Results

The content of antioxidant compounds, such as total phenolics and anthocyanins, was quantified. The total phenolic compounds in the aqueous extract of* P. trunciflora* were 1201.67 ± 33.29 mg GAE/100 g, while the anthocyanins content was 175.33 ± 11.80 mg cyanidin 3-*O*-glucoside equivalents/100 g.

**Figure 1:**
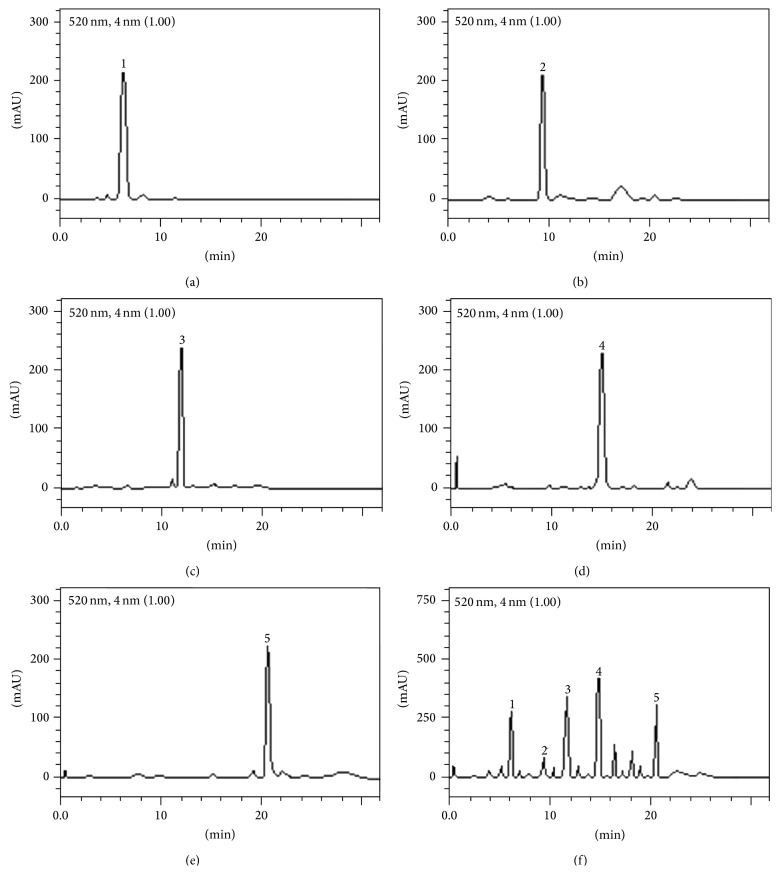
Representative high performance liquid chromatography profiles of the standards cyanidin chloride (a), malvidin chloride (b), delphinidin 3-*O*-glucoside chloride (c), cyaniding 3-*O*-glucoside chloride (d), malvidin 3-*O*-glucoside chloride (e), and PT (f).

The HPLC fingerprinting of the* P. trunciflora* aqueous extract (Figure 1) revealed the presence of anthocyanins. We identified cyanidin (Rt = 6.41 min; peak 1), malvidin (Rt = 9.73 min; peak 2), delphinidin 3-*O*-glucoside (Rt = 11.94 min; peak 3), cyanidin 3-*O*-glucoside (Rt = 15.08; peak 4), and malvidin 3-*O*-glucoside (Rt = 20.57 min; peak 5). The composition of anthocyanins (mg/g) in the* P. trunciflora* aqueous extract was cyanidin (16.2 ± 0.01), malvidin (3.5 ± 0.02), delphinidin 3-*O*-glycoside (24.1 ± 0.03), cyanidin 3-*O*-glycoside (27.6 ± 0.03), and malvidin 3-*O*-glycoside (17.1 ± 0.02).

**Figure 2:**
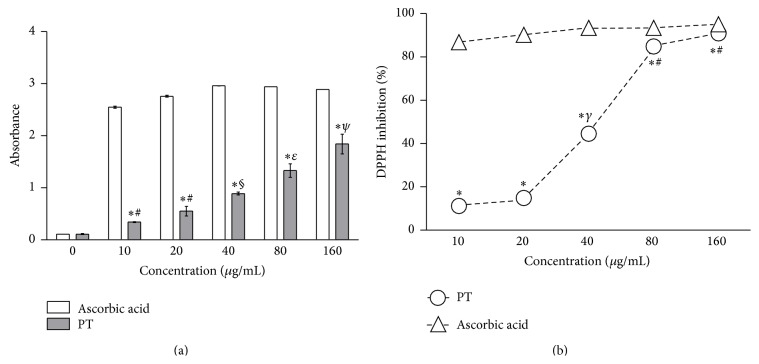
Ferric reducing antioxidant power (FRAP (a)) and DPPH radical scavenger activity (b) of PT. The results are expressed as the mean ± S.E.M. *n* = 3. (a) ^*∗*^Different from ascorbic acid solution at the same concentration. _ _^*∗*^ *p* < 0.05; Student's *t*-test. Different symbols represent different results within the PT group (*p* < 0.01, ANOVA/Tukey). (b) ^*∗*^Different from DPPH radical scavenger activity of ascorbic acid solution at the same concentration. _ _^*∗*^ *p* < 0.001, Student's *t*-test. Different symbols represent different results within the PT group (*p* < 0.05, ANOVA/Tukey).

A one-way ANOVA revealed that PT showed significant reducing power beyond a concentration of 10 *μ*g/mL (Figure 2(a)). However, the ferric reducing power of ascorbic acid was higher than that shown by PT in all concentrations evaluated, as evident from Student's *t* test.  Figure 2(b) shows the DPPH radical scavenging antioxidant activity. Although the DPPH radical scavenging ability of PT was lower than that of the ascorbic acid solution, it was remarkable at all evaluated concentrations. The calculated IC_50_ value for PT was 42.2 *μ*g/mL, compared to a value of 0.04 *μ*g/mL for ascorbic acid. At 10 and 20 *μ*g/mL, PT inhibited between 11 and 14% of DPPH; at 40 *μ*g/mL, the inhibition increased to 50% and exceeded 80% at concentrations of 80 and 160 *μ*g/mL.

**Figure 3:**
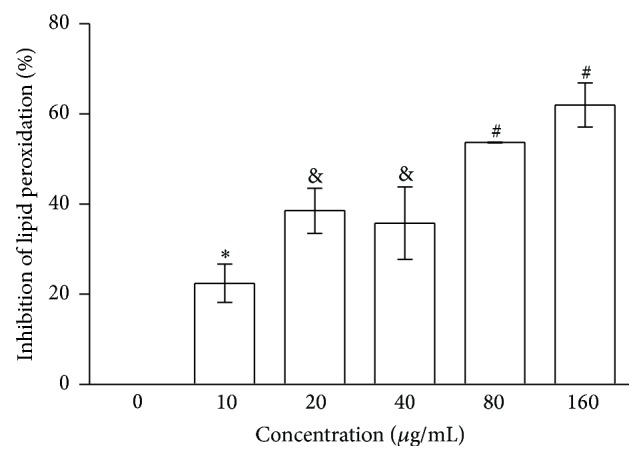
Inhibition of Fenton reaction-induced lipid peroxidation of PT. The results are expressed as the means ± S.E.M. *n* = 5.  ^*∗*^Different symbols represent different results within the PT group (*p* < 0.05, ANOVA/Tukey).

The extract inhibited the lipid peroxidation in a homogenate of mouse brain at all concentrations (Figure 3). At 10 *μ*g/mL, PT inhibited approximately 20% of lipid peroxidation, and the inhibition increased to 40% at 20 and 40 *μ*g/mL and reached 60% at concentrations of 80 and 160 *μ*g/mL. The calculated IC_50_ value for PT was 70.4 *μ*g/mL.

**Figure 4:**
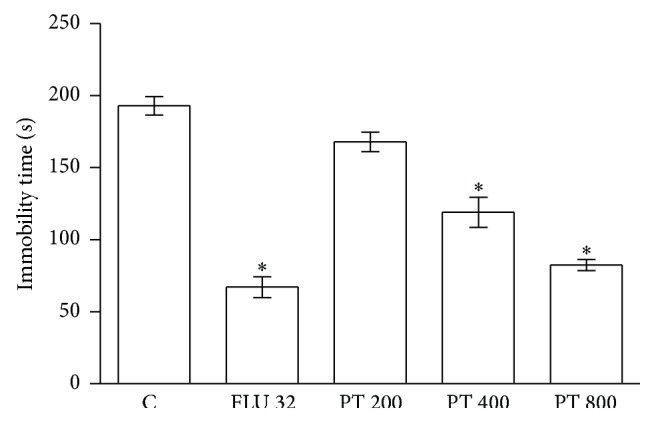
Effects of PT (200, 400, and 800 mg/kg,* p.o*.) and fluoxetine (32 mg/kg,* i.p*.) in the TST. Each column represents the mean ± S.E.M. *n* = 7–10.  _ _^*∗*^ *p* < 0.0001  × saline. ANOVA/ Tukey.

The results in Figure 4 show the effects of PT (200, 400, and 800 mg/kg,* p.o*.) and fluoxetine (32 mg/kg,* i.p*.) during the tail suspension test in mice. PT significantly reduced the immobility time in the TST (400 and 800 mg/kg,* p.o*., *F*_4,40_ = 48, *p* < 0.0001). Fluoxetine significantly reduced the immobility time in the TST. The PT (800 mg/kg,* p.o*.) was compared with fluoxetine. The spontaneous locomotion of groups treated with PT did not differ from the controls (data not shown).

**Figure 5:**
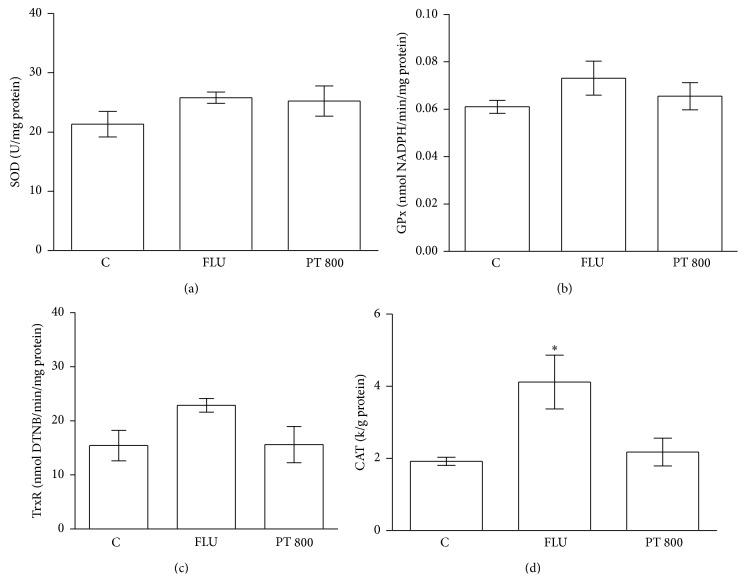
Effects of PT (800 mg/kg,* p.o*.) and fluoxetine (32 mg/kg,* i.p*.) treatment on antioxidant enzyme activities SOD (a), GPx (b), TrxR (c), and CAT (d) in homogenate of brain mice. The results are expressed as the means ± S.E.M. *n* = 6. _ _^*∗*^ *p* < 0.05  × saline. ANOVA/Tukey.

Figure 5 presents the effect of PT (800 mg/kg,* p.o*.) and fluoxetine (32 mg/kg,* i.p*.) administration on the antioxidant enzyme activities in the homogenate of mouse brains. PT and fluoxetine did not result in significant changes in the SOD (Figure 5(a)), GPx (Figure 5(b)), and TrxR (Figure 5(c)) activities compared to the controls; fluoxetine significantly increased the CAT (Figure 5(d)) activity compared to the controls.

**Figure 6:**
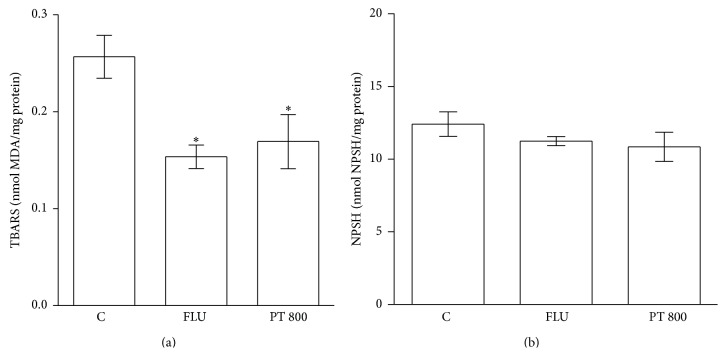
Effects of PT (800 mg/kg,* p.o*.) and fluoxetine (32 mg/kg,* i.p*.) on lipid peroxidation (a) and nonprotein thiol groups (NPSH) level (b). The results are expressed as the means ± S.E.M. *n* = 6.  _ _^*∗*^ *p* < 0.05  × saline. ANOVA/Tukey.

Figure 6 shows the effect of PT (800 mg/kg,* p.o*.) and fluoxetine (32 mg/kg,* i.p*.) on the lipid peroxidation and level of nonprotein thiol groups (NPSH) in the homogenate of mouse brains. Both the PT extract and the fluoxetine attenuated lipid peroxidation (Figure 6(a)). The levels of nonprotein thiol in the PT extract and fluoxetine (Figure 6(b)) groups did not differ from that of the control.

## 4. Discussion

Depression has been associated with lowered concentrations of several endogenous antioxidant compounds, such as coenzyme Q10, vitamins C and E, or antioxidant enzymes, such as GPx [29]. In addition, ROS and RNS have been shown to modulate neurotransmitter systems involved in the neurobiology of depression [30]. In this context, this study intended to evaluate the antidepressant-like effect of a* P. trunciflora* (PT) aqueous extract using the TST. Moreover, considering that jaboticaba species are rich in flavonoids and related polyphenols [31], the antioxidant effects of PT were evaluated by* in vitro* and* ex vivo* assays.

Our results showed for the first time that oral PT (400 and 800 mg/kg) had antidepressant-like activity in the TST. This effect was dose related (*r* = −0.84, *p* < 0.001, Pearson correlation analysis). Furthermore, the effect of PT (800 mg/kg) was comparable to that of the antidepressant fluoxetine (32 mg/kg), a selective serotonin reuptake inhibitor. To avoid false positives in the TST, our results showed that PT treatment did not alter locomotor activity in the open field test (Figure  1S, in Supplementary Material available online at http://dx.doi.org/10.1155/2015/601503).

Although the mechanisms of the antidepressant-like activity of PT remain unclear, the bioactive compounds currently identified as well as their antioxidant properties may be involved in this effect. Flavonoids, such as anthocyanins, stand out among the major classes of phenolic compounds of plants [32]. The cyanidin-3-*O*-glycoside (peak 4) was the dominant anthocyanin present in our extract. Other anthocyanins, such as delphinidin 3-*O*-glucoside and cyanidin- 3-*O*-glucoside, were also detected. Importantly, the antioxidant effects of these compounds have been described in the literature [33]. Data of linear regression revealed a substantial contribution of TPC and TMA for the reducing antioxidant power of PT assessed by FRAP method (*R* = 0.96 and *R* = 0.97, *p* = 0.00001, resp.; see Figures  2S-3S, supplementary data). TPC and TMA also contributed to % inhibition of DPPH and % inhibition of lipid peroxidation (*R* = 0.83 and *R* = 0.93, resp.), as demonstrated by nonlinear regression (see Figures  4S–7S, supplementary data).

The FRAP assay measures the ability of an antioxidant substance to donate one electron [18]. Because the antioxidant activity of a substance correlates with its reducing properties, the reduction of the 2,4,6-tripyridyl-s-triazine-Fe(III) complex due to PT indicates the presence of compounds that can donate electrons, such as phenolic compounds. Accordingly, the antioxidant properties of the* Syzygium cumini* fruit skin may in part be due to the antioxidant vitamins, tannins, phenolics, and anthocyanin compounds present in the fruit [34].

The reducing power of PT was corroborated by the DPPH radical scavenging assay, which also evaluates the ability of antioxidants to transfer a single electron. This affirmation is based on the fact that both the reducing and the scavenging DPPH abilities of the extract were observed in the entire evaluated concentration range (10–160 *μ*g/mL). Therefore, these results strongly suggest that the DPPH radical scavenging capacity of PT is related to its reducing properties, as evidenced in the FRAP assay. Conversely, PT could not remove H_2_O_2_ or O_2_^•−^ nor avoid the H_2_O_2_-induced oxidation of GSH (data not shown).

Lipid peroxidation is an index of oxidative stress and may result in damage to components of the cell membrane, which may lead to calcium influx and cell death. Lipid peroxidation is associated with several diseases, including neurodegenerative disorders [35], and antioxidants may protect against lipid peroxidation by scavenging the free radicals [36]. The* in vitro* results of the current study showed that PT inhibited the lipid peroxidation at all concentrations. This protective effect suggests that other possible mechanisms of action of the antioxidant activity are associated with the ability of this extract to scavenge the hydroxyl (^•^OH) radical. Interestingly, PT (800 mg/kg) also attenuated lipid peroxidation when administered to mice. This protection was similar to that observed for fluoxetine (32 mg/kg) and occurred at a dose that showed an antidepressant-like effect. Fluoxetine decreased lipid peroxidation probably due to the increased CAT activity, which removes H_2_O_2_ to reduce its availability for the formation of the ^•^OH radical. Similarly, fluoxetine exerted a restorative action on the oxidative effects in the peripheral defense cells of animals submitted to the restraint stress model, which was also associated with enhanced endogenous antioxidant defenses (CAT and SOD) and the restoration of GSH levels [37]. Oxidative damage to lipids and decreased antioxidant enzyme activity have been reported in patients with major depressive disorder [38], and preclinical studies have suggested that antioxidants may have antidepressant properties [39]. Taking into account these findings, the inhibition of lipid peroxidation by PT as well as the ability of PT to scavenge free radicals strongly suggests a link between the antioxidant activity and the antidepressant-like effects observed here.

The present study showed the* in vitro* and* ex vivo* antioxidant and antidepressant-like effects of PT in mice. These antioxidant properties might be related to the antidepressant-like activity of* Plinia trunciflora*.

## Supplementary Material

The Figure 1S shows the effects of PT (200, 400 and 800 mg/kg, p.o.) and fluoxetine (32 mg/kg, i.p.) on the number of crossings in the open field test. Each column represents the mean ± S.E.M. n = 7—10. ANOVA/Tukey.
Fig. 2S. Linear correlation graph for TPC and FRAP. Coefficient of correlation (R)= 0.96, coefficient of determination (R^2^)= 0.92.
Fig. 3S. Linear correlation graph for TMA and FRAP. Coefficient of correlation (R) = 0.97, coefficient of determination (R^2^) = 0.94.
The Figures 4S–7S show the contribution of TPC and TMA for % inhibition of DPPH and % inhibition of lipid peroxidation (R=0.83 and R=0.93, respectively), as demonstrated by nonlinear regression.
