# Supplementary material for: Antidepressant-Like and Antioxidant Effects of Plinia trunciflora in Mice
Source: Evid Based Complement Alternat Med. 2015 Jul 2;2015:601503. doi: 10.1155/2015/601503 (PMC4503558; doi:10.1155/2015/601503)
Supplement: Supplementary file 1 — The Figure 1S shows the effects of PT (200, 400 and 800 mg/kg, p.o.) and fluoxetine (32 mg/kg, i.p.) on the number of crossings in the open field test. Each column represents the mean ± S.E.M. n = 7—10. ANOVA/Tukey. Fig. 2S. Linear correlation graph for TPC and FRAP. Coefficient of correlation (R)= 0.96, coefficient of determination (R2)= 0.92. Fig. 3S. Linear correlation graph for TMA and FRAP. Coefficient of correlation (R) = 0.97, coefficient of determination (R2) = 0.94. The Figures 4S–7S show the contribution of TPC and TMA for % inhibition of DPPH and % inhibition of lipid peroxidation (R=0.83 and R=0.93, respectively), as demonstrated by nonlinear regression. [file 601503.f1.docx]

**Supplementary materials for the manuscript entitled “Antidepressant-like and antioxidant effects of *Plinia trunciflora* in mice” by Sacchet et al.**

**

**

**Fig. 1S.** Effects of PT (200, 400 and 800 mg/kg, p.o.) and fluoxetine (32 mg/kg, i.p.) on the number of crossings in the open field test. Each column represents the mean ± S.E.M. n=7-10. ANOVA/ Tukey.





**Fig. 2S.** Linear correlation graph for TPC and FRAP. Coefficient of correlation (R)= 0.96, coefficient of determination (R^2^)= 0.92.





**Fig. 3S.** Linear correlation graph for TMA and FRAP. Coefficient of correlation (R) = 0.97, coefficient of determination (R^2^) = 0.94.





**Fig. 4S.** Nonlinear regression graph for TPC and % inhibition of DPPH. Coefficient of correlation (R) = 0.83, coefficient of determination (R^2^) = 0.70.





**Fig. 5S.** Nonlinear regression graph for TMA and % inhibition of DPPH. Coefficient of correlation (R) = 0.83, coefficient of determination (R^2^) = 0.70.





**Fig. 6S.** Nonlinear regression graph for TPC and % inhibition of lipid peroxidation. (R) = 0.93, coefficient of determination (R^2^) = 0.88.





**Fig. 7S.** Nonlinear regression graph for TMA and % inhibition of lipid peroxidation. (R) = 0.93, coefficient of determination (R^2^) = 0.88.
